# Commercially Available Smartphone Apps to Support Postoperative Pain Self-Management: Scoping Review

**DOI:** 10.2196/mhealth.8230

**Published:** 2017-10-23

**Authors:** Chitra Lalloo, Ushma Shah, Kathryn A Birnie, Cleo Davies-Chalmers, Jordan Rivera, Jennifer Stinson, Fiona Campbell

**Affiliations:** ^1^ The Hospital for Sick Children Department of Child Health Evaluative Sciences Toronto, ON Canada; ^2^ Department of Anesthesiology and Perioperative Medicine University of Western Ontario London, ON Canada; ^3^ Lawrence S. Bloomberg Faculty of Nursing University of Toronto Toronto, ON Canada; ^4^ Department of Anesthesia and Pain Medicine The Hospital for Sick Children Toronto, ON Canada; ^5^ Department of Anesthesia University of Toronto Toronto, ON Canada

**Keywords:** pain, postoperative, smartphone, mobile applications, review, pain management, self care

## Abstract

**Background:**

Recently, the use of smartphones to deliver health-related content has experienced rapid growth, with more than 165,000 mobile health (mHealth) apps currently available in the digital marketplace. With 3 out of 4 Canadians currently owning a smartphone, mHealth apps offer opportunities to deliver accessible health-related knowledge and support. Many individuals experience pain after surgery, which can negatively impact their health-related quality of life, including sleep, emotional, and social functioning. Smartphone apps that provide remote real-time monitoring and symptom management have the potential to improve self-management skills in patients experiencing postoperative pain. Increased confidence and practice of self-management skills could contribute to decreased postoperative pain and reduce risk of developing persistent pain. Published reviews of general pain self-management apps demonstrate a lack of evidence-based content, theoretical grounding, and health care professional involvement. However, no review to date has focused on the app marketplace specific for individuals with postoperative pain.

**Objective:**

The aim of this study was to characterize and critically appraise the content and functionality of commercially available postoperative pain self-management apps.

**Methods:**

An electronic search and extraction was conducted between December 2016 and March 2017 of the official Canadian app stores for the three major smartphone operating systems (iPhone operating system [iOS], Android, and Windows). Stores were searched separately using predetermined search terms. Two authors screened apps based on information provided in the public app description. Metadata from all included apps were abstracted into a standard spreadsheet. Two authors verified the data with reference to the apps and downloaded apps themselves. The content and functionality of each app as it pertained to postoperative pain self-management was rated.

**Results:**

A total of 10 apps met the inclusion criteria. All included apps were designed exclusively for the Android platform. Education was the most common self-management feature offered (8/10, 80%), with none of the apps offering features related to goal setting or social support. Overall, no single app was comprehensive in terms of pain self-management content. Five (50%) apps reported the involvement of a health care provider in their development. However, not a single app involved end users in their development, and none of the apps underwent scientific evaluation. Additionally, none of the apps were designed for use in pediatric patients.

**Conclusions:**

Currently available postoperative pain apps for patients lack evidence-based content, goal setting, and social support functions. There is a need to develop and test comprehensive theory-based apps to support patients with pain self-management care following surgery.

## Introduction

### Background

More than 80% of patients experience acute postoperative pain after surgery, of whom approximately 75% report their pain intensity as moderate, severe, or extreme [1,2]. Unrelieved or undertreated acute postoperative pain can delay remobilization, lead to increased opioid use and related side effects, and negatively impact all aspects of health-related quality of life [3-5]. Greater confidence in one’s ability to control pain is referred to as pain coping self-efficacy. Improvements in pain coping self-efficacy may contribute to decreased postoperative pain and reduced risk for developing persistent postoperative pain, an expensive and debilitating health problem [6,7]. Thus, the prevention or minimization of postoperative pain is critically important.

Remote real-time monitoring and symptom management support for postoperative pain patients is a relatively new notion made possible by technological advances. Smartphones, for instance, are now widely used across the life span [8,9]. Smartphones have the potential to empower people with postoperative pain who are at increased risk for undertreatment of pain and disruption in activities of daily living [10]. Smartphones can support self-management by (1) improving self-monitoring of pain and other symptoms in everyday environments (eg, home, hospital settings), (2) promoting pain self-efficacy and appropriate self-care (eg, adherence to prescribed medications, cognitive-behavioral pain coping strategies), and (3) minimize barriers to optimal pain treatment (eg, lack of transportation to tertiary care centers) [11,12].

### Existing Reviews of Pain Management Apps

There are a growing number of generalized pain self-management smartphone apps available for users to download on their personal mobile devices. In a 2011 scoping review, Rosser and Eccleston identified 111 such apps [13]. A majority were designed for the iPhone (iPhone operating system, iOS) platform, with fewer being available for Android or BlackBerry operating systems. The primary app functions included education skills training (50.5%; 56/111), self-monitoring (eg, pain diary, 26.1%; 29/111), and relaxation training (21.6%; 24/111). Importantly, 85.6% (95/111) of the identified apps did not report the involvement of health care professionals in their design or evaluation of content. None of the apps were focused on postoperative pain [13]. In 2012, Reynoldson and colleagues conducted an updated search of the iOS and Android stores [14]. This search only included apps with a primary self-monitoring function (ie, allowing patients to track their pain episodes). Apps were excluded if their intended use was limited to pain from specific conditions such as arthritis, inflammatory bowel disease, or migraine. The authors identified 12 eligible apps, which then underwent content analysis and usability testing. Their review identified a lack of user and clinician engagement in pain app development, as well as variation in app quality [14]. In 2015, Lalloo and colleagues conducted an updated scoping review of available pain management apps [15]. A total of 279 apps met the inclusion criteria. Pain self-care skill support was the most common self-management function (77.4%; 216/279). Apps also purported providing patients with the ability to engage in pain education (45.9%; 128/279), self-monitoring (19.0%; 53/279), social support (3.6%; 10/279), and goal setting (0.7%; 2/279). No apps were comprehensive in terms of pain self-management, with the majority of apps including only a single self-management function (58.8%; 164/279). Additionally, only 8.2% (23/279) of the apps included a health care professional in their development, not a single app provided a theoretical rationale, and only 1 app underwent scientific evaluation [15].

### Objectives

To our knowledge, no scoping review to date has focused on apps for postoperative pain management. In addition, no scoping reviews to date have sought to identify apps for both pediatric and adult users. Therefore, the objectives of this study were to (1) characterize the current field of patient-focused pain self-management apps across all major smartphone platforms in Canada; (2) critically appraise the content and functionality of these apps, including self-care skill support, pain education, self-monitoring, social support, and goal setting; and (3) identify gaps in the field to guide future development of an app to support individuals with postoperative pain.

## Methods

An electronic search and extraction was conducted between December 2016 and March 2017 of the official Canadian app stores for the 3 major smartphone operating systems: iOS (iTunes App Store), Android (Google Play Store), and Windows (Windows Store). The entire stores were searched separately using predetermined search terms (ie, no restrictions related to store subcategories such as “health and wellness” were imposed). The search terms were as follows: “Pain, Surgery,” “Pain, Post-Surgery,” “Pain, Post-Surgical,” “Pain, Operative,” “Pain, Operation,” “Pain Management, Surgery,” “Pain Management, Post-Surgery,” “Pain Management, Post-Surgical,” “Pain Management, Operative,” “Pain Management, Operation.”

No limits were imposed related to language or date of app publication. Given that the process for app indexing exhibited variability across app stores, a calibration exercise was conducted before app selection in order to verify our ability to detect postsurgical pain apps. Specifically, we tested our search criteria for the ability to find postsurgical pain apps that were known to be currently available in the app stores. In all cases, our search criteria identified the apps we expected to find.

### Screening and Selection of Apps

Apps were included in the study if the primary intended user of the app was a person undergoing surgery, and a stated goal of the app was to provide education, tools, or advice related to managing pain after surgery. Apps were excluded if they focused only on the services offered by specific hospitals because they were intended as advertisements for these sites. These *clinic apps* typically included only lists of the pain management services and personnel available at the hospital and directions to the clinic rather than self-management programming. Apps were also excluded if they were classified as *e-books* by the respective app store or were judged by the reviewers as such. An *e-book* was defined as an app that did not provide any additional content or functionality beyond a textbook (eg, written content identical to a book). Authors performed app selection independently and all discrepancies regarding selection were resolved through discussion with a third author. Apps were screened based on the information provided in the *app description* section of each respective app store. Apps that met all screening criteria were downloaded and reviewed to confirm eligibility, and then data abstraction and content assessment were completed.

### Data Abstraction

Metadata from all included apps were abstracted into a Microsoft Excel spreadsheet. Abstracted metadata included app name, URL, app description, customer rating on the day of store search, number of installations, and price. A systematic approach to data abstraction was used. Specifically, 2 authors (CDC and JR) abstracted all data and 2 others (CL and US) verified the data with reference to the app website and the downloaded apps themselves.

### Content Assessment

To assess the comprehensiveness of app self-management content, the following criteria were used: (1) having a postoperative pain tracking function, (2) ability to set goals related to improving pain and functioning, (3) provision of skills training related to specific pain self-care strategies, (4) provision of social support, (5) provision of education related to surgery, and (6) provision of education related to pain after surgery. For each criterion, we assessed coverage as either *present* or *absent*. Similar criteria were used in our 2015 published scoping review [15] of general pain self-management apps. This list is based on the features of pain self-management programs that have been examined for effectiveness in scientific trials [16-19].

We also assessed whether a regulated health care professional had provided clinical expertise related to development of the app content and function. An app was required to reference the involvement of a health care professional listed in the Ontario Regulated Health Professions Act [20] to meet this criterion. This list contains 26 health care regulatory colleges to whom the Ontario government has endowed the capacity to regulate the practices of corresponding health care professionals to ensure safe and ethical patient care. The list includes professions such as physicians, nurses, and physiotherapists. If an app claimed the input of a profession that was not listed in the Act in its development, we assessed the health care professional involvement as *absent*. The involvement of end users (eg, surgery patients) in app development was also documented as *present* or *absent*. Apps were reviewed to determine whether they were designed for pediatric (aged 18 years or younger) or adult users.

The app store descriptions were also screened for any reference to the app being part of formal scientific research. The app description and corresponding developer website (if available) were reviewed to determine whether any specific theoretical framework had been used to guide the development of app content. Finally, large publicly accessible scientific literature databases (ie, NCBI PubMed and Google Scholar) were searched using the app name as a query for any published research related to the app. The MyHealthApps website was also reviewed as per the methods of de la Vega and Miro [21]. Descriptive statistics were used to summarize the results of the content assessment.

## Results

### App Screening

**Figure 1:**
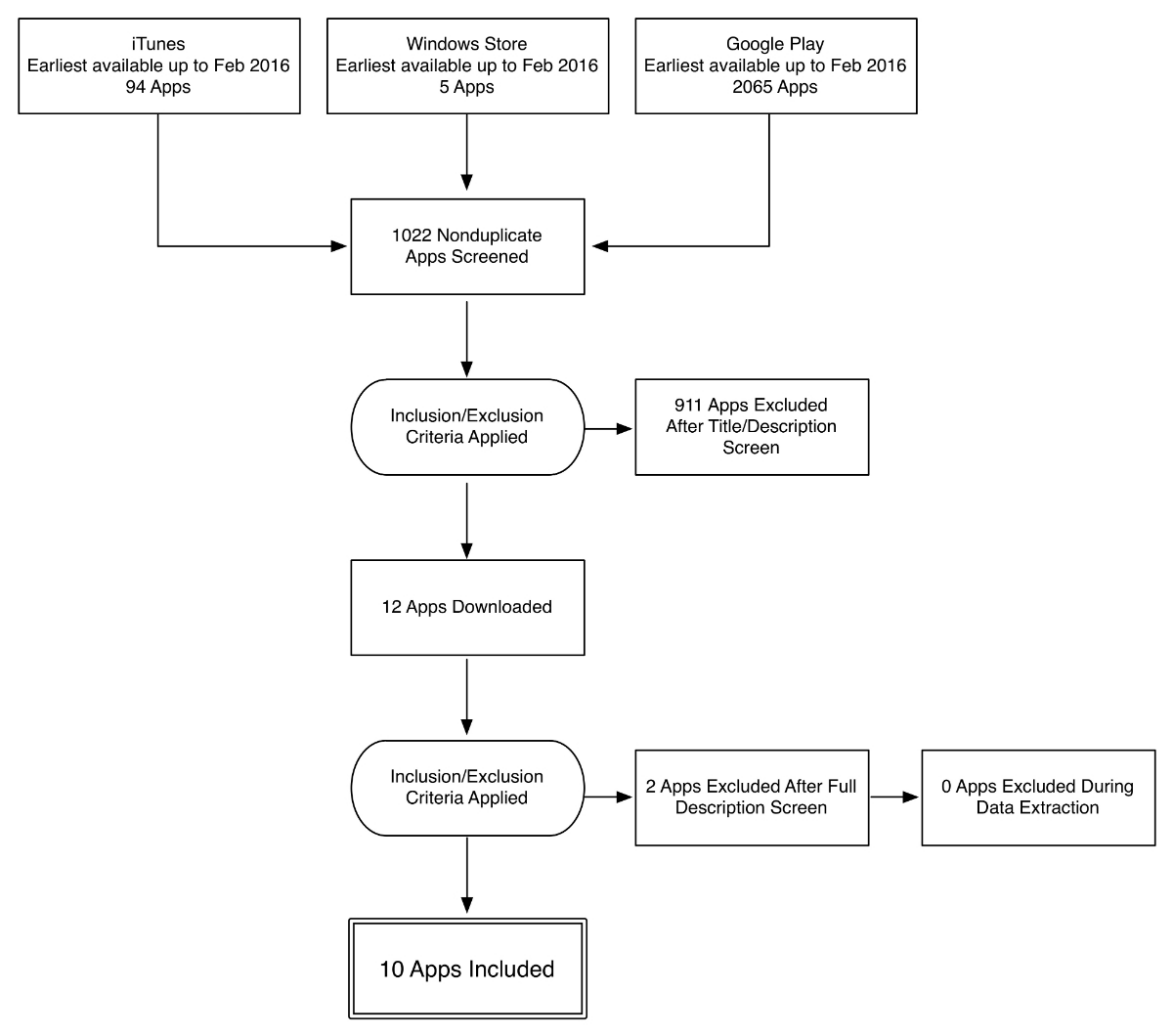
PRISMA (Preferred Reporting Items for Systematic Reviews and Meta-Analyses) flowchart of app review process.

Our search strategy across iTunes, Windows, and Google Play app stores identified a total of 1019 apps (923 Android, 93 iPhone, and 3 Windows), which were screened according to the inclusion and exclusion criteria. Only 10/1019 apps (0.98% of total screened) met all inclusion criteria and were included in the analysis (see Figure 1 for PRISMA [Preferred Reporting Items for Systematic Reviews and Meta-Analyses] flowchart) [22]. All included apps were designed exclusively for the Android platform (see Table 1 for included apps).

### Self-Management Content of Reviewed Apps

**Figure 2:**
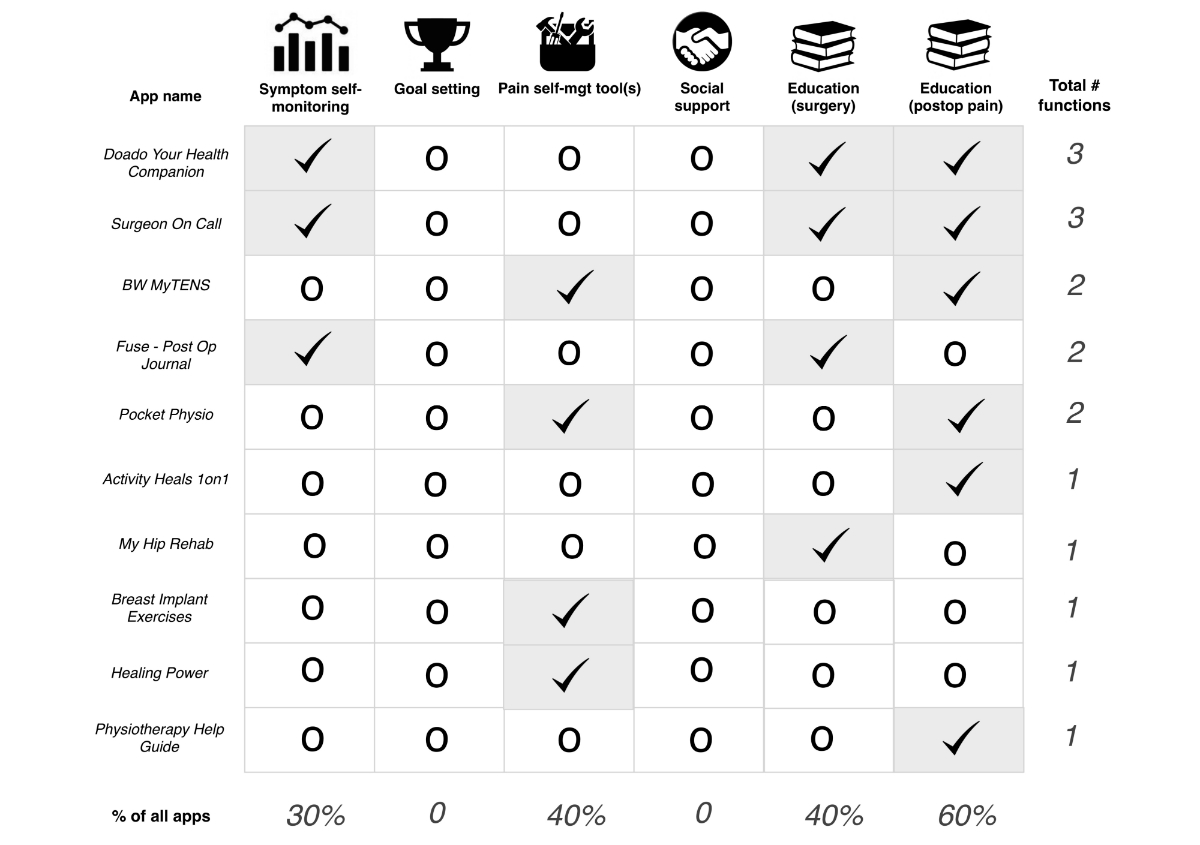
Self-management content across reviewed apps.

Education related to postoperative pain was the most common self-management feature offered across the identified apps. This educational content largely focused on self-care advice following surgery, particularly physiotherapy exercises to support return to mobility. However, there was no specific content related to emotional factors such as anxiety or catastrophizing related to surgery. As well, none of the apps offered features related to goal setting or social support. Overall, no single app was comprehensive in terms of pain self-management content. Additionally, no apps were designed specifically for pediatric patients. See Figure 2 for a summary of the self-management components across all reviewed apps.

**Table 1:** Postoperative pain apps included in the review.

#	App	Developer
1	Activity Heals 1on1	Elevenity
2	Breast Implant Exercises	Chicago Plastic Surgery
3	BW MyTENS	Visiomed Lab
4	Doado Your Health Companion	Alex Spriet
5	Fuse—Post Op. Journal	Imbas Solutions
6	Healing Power	Phil Shapiro
7	My Hip Rehab	App Squid
8	Physiotherapy Help Guide	Creativity Knowledge App
9	Pocket Physio	Care UK HC
10	Surgeon on Call	Phoenix Medical Consulting

### App Cost, Customer Ratings, and Number of Installs

**Figure 3:**
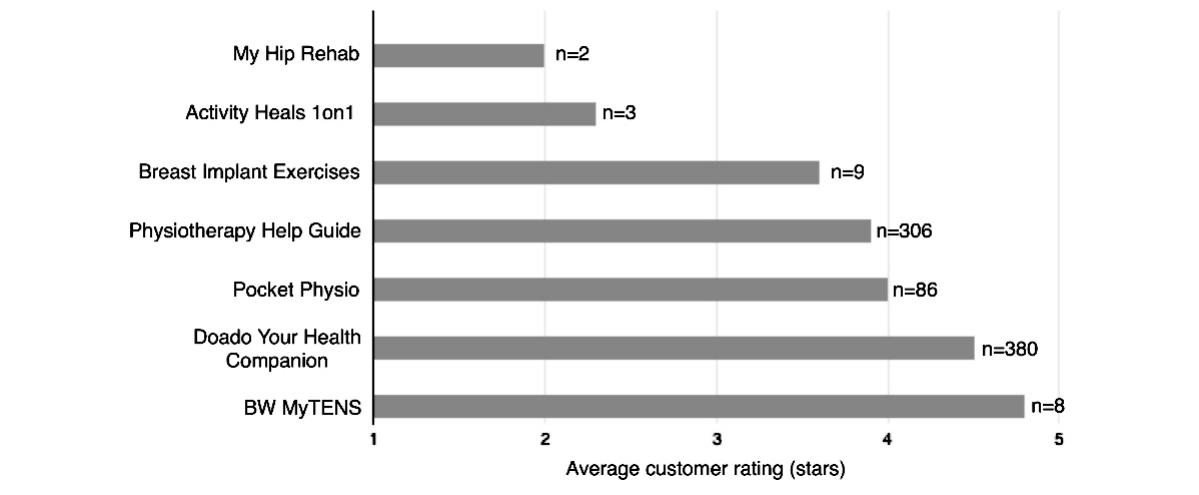
Customer-generated app ratings. *No customer ratings, or less than 2 ratings, were available for three apps: Fuse – Post-Op Journal, Surgeon on Call, and Healing Power.

Most apps (8/10, 80%) were free to download with the exceptions of *Surgeon on Call* and *My Hip Rehab*, which were associated with charges of Can $0.99 and Can $10.22, respectively. App store customers had the option of rating each app on a scale ranging from 1 to 5 stars (no anchors provided). There was a high variability in the number of users who chose to rate each app (range of 2-380 raters per app). As seen in Figure 3, the average customer rating ranged from 2 to 4.8 stars. However, fewer than 10 customers reviewed most (7/10, 70%) of the apps. The number of app installs ranged from as low as 10 to as high as 100,000 across apps (see Table 2).

**Table 2:** App installations. Categories provided by Android Google Play Store. Data collected on April 26, 2017.

Number of installs	Apps
10-50	Surgeon on Call; My Hip Rehab
100-500	Fuse–Post Op. Journal; Healing Power
500-1000	Activity Heals 1on1; BW MyTENS
1000-5000	Breast Implant Exercises
5000-10000	Pocket Physio
10,000-50,000	Doado Your Health Companion
50,000-100,000	Physiotherapy Help Guide

### Involvement of Health Care Providers, End Users, and Scientific Evaluation

Overall, 5/10 (50%) apps reported the involvement of a health care provider in their development. In summary, *Doado Your Health Companion* and *Pocket Physio* involved a physiotherapist, while *Breast Implant Exercises* and *Healing Power* involved a physician, and *Surgeon on Call* involved a specialist (surgeon). The other apps did not report any involvement of health care professionals in their development. None of the apps reported involvement of end users (eg, surgical patients) in their development. No evidence was found indicating that any of the apps had been scientifically evaluated.

## Discussion

### Principal Findings

This review demonstrates the paucity of high-quality apps that are commercially available to support self-management of postoperative pain for either adult or pediatric users. All included apps were designed exclusively for the Android platform. Education was the most common self-management feature offered (8/10, 80%), while none of the apps offered features related to goal setting or social support. Overall, no single app was comprehensive in terms of pain self-management content. Five (5/10, 50%) apps reported the involvement of a health care provider in their development. However, not a single app involved end users in their development and none of the apps underwent scientific evaluation. Additionally, none of the apps were designed for use in pediatric patients.

Customer ratings for the apps were moderate on average (mean 3.6, standard deviation 1.1). However, it is important to highlight that very few users chose to provide an app rating. For instance, while *Pocket Physio* reported between 5000 and 10,000 installs, only 86 users provided a rating (0.86-1.7%). No ratings, or less than the required minimum of 2 ratings, were provided for 30% (3/10) of the apps. All of the identified apps were designed exclusively for Android devices with no interoperability for iOS and Windows devices. This finding is in contrast to the 2015 systematic review of generic pain apps, where the majority of identified apps were designed for the iOS platform [15].

The adequacy of existing apps can be assessed relative to recently published clinical practice guidelines for evidence-based management of postoperative pain across the life span [23]. Relevant recommendations include the delivery of individually tailored preoperative education and perioperative pain management planning, the use of validated pain assessment tools to track response to pain interventions and inform treatment adjustments as needed, as well as the use of evidence-based, nonpharmacological pain management (eg, cognitive behavioral strategies, physical modalities) in conjunction with indicated pharmacological modalities [23]. Compared against these guidelines, none of the reviewed apps uniquely fulfill all recommended criteria of education (surgery and pain), symptom self-management, and pain self-management strategies. Furthermore, none of the apps offer the ability to prespecify a postoperative pain management plan or to consider the relevance of managing presurgical pain to postsurgical outcomes.

A complex myriad of surgical, psychological, socioenvironmental, and patient-related risk factors have been shown to influence postsurgical pain experience [24]. Pre- and postsurgical psychological factors associated with increased pain include anxiety, depression, low self-efficacy, and the tendency to catastrophize about pain [24-26]. Recent reviews and meta-analyses identify psychological treatments delivered preoperatively, postoperatively, or both, to effectively reduce postoperative pain [23,27]. Many of these strategies, such as distraction, relaxation, and guided imagery techniques, can be easily delivered via mHealth platforms. The ability of self-management apps to target psychosocial risk factors is plausible, given existing evidence supporting the use of smartphones to effectively deliver interventions to reduce symptoms of anxiety and depression [28], particularly when strategies are available in the moment when people are engaged in their everyday lives [29].

Despite the focus of many apps on physical strategies in recovery from surgery, none included goal setting. Goal setting is a critical function in smartphone apps targeting increased physical activity [30]. In addition to increasing risk for postsurgical pain, depression, anxiety, and low self-efficacy are also identified as barriers to adherence to physiotherapy treatment [31]. Apps that are designed to pair psychological and physical pain self-management strategies with goal setting may be particularly effective for reducing pain and enhancing postsurgical outcomes.

Concurring with the findings of earlier app reviews [13,15,32], this study demonstrates that existing postoperative pain apps lack empirical evidence of evaluation. This finding continues to raise concerns about the trustworthiness and effectiveness of commercially available apps for helping individuals to manage their postoperative pain. In contrast to previous reviews, 50% (5/10) of the identified postoperative pain apps reported inclusion of a health care provider in their development. While this is a positive development, it would be informative for app developers to more explicitly describe the nature of provider input.

Moreover, none of the apps explicitly involved end users (people with postoperative pain) in their development. It has been recommended that patient-focused apps be developed as per a user-centered design (UCD) approach [33]. UCD is “characterized by a focus on the user, and on incorporating the user’s perspective in all stages of the design process” [34]. Application of UCD has been associated with improved user acceptance, satisfaction, and engagement [35]. Given the limitations of existing apps, it is important that future app development to support postoperative pain management adopt the UCD principles.

### Limitations

Given that the Web-based app searches were conducted within Canada, the results reflect the Canadian app marketplace. Due to the geographic search restrictions of the online app stores, this limitation also applies to other published app reviews (eg, Rosser and Eccleston [13] and Reynoldson et al [14] focused on the United Kingdom; Lalloo et al [15] focused on Canada). Thus, there may be differences in the commercially available postoperative pain apps in other countries. Additionally, while the Google Play Store (which contained all included apps) allows users to rate their app experience, their 1 to 5 scale does not have defined anchors. This lack of definition limits our ability to interpret the factors that are driving customer ratings. Furthermore, since app customers are not required to provide ratings, there is a high frequency of missing rating data in comparison with the reported number of installs.

### Conclusions

Currently available postoperative pain apps are characterized by a lack of evidence-based content, goal setting, and social support functions and are not targeted to pediatric users. There is a need to develop and evaluate comprehensive, theory-based apps to better support patients with pain self-management care following surgery.

## Abbreviations

mHealth: mobile health
PRISMA: Preferred Reporting Items for Systematic Reviews and Meta-Analyses
UCD: user-centered design
